# Distal Humerus Fracture Malunion in Adults: A Case Report and a Review of the Literature

**DOI:** 10.1155/2022/6041577

**Published:** 2022-04-29

**Authors:** Ioannis M. Stavrakakis, Zisis Ntontis, Olga Kastritsi, Constantinos Chaniotakis, Kalliopi Alpantaki, Grigorios Kastanis

**Affiliations:** Department of Orthopaedics and Traumatology, Venizeleio and Pananio General Hospital of Heraklion Crete, Greece

## Abstract

**Introduction:**

Neglected distal humerus fractures are rare injuries, which the orthopaedic surgeon will not deal many times in his career. We present a case of a young patient with such an injury, who was treated with a corrective osteotomy and fixation, resulting in a very good outcome. This case report highlights the importance of surgical intervention for distal humerus fracture malunion. A narrative review of the literature regarding this topic is presented as well. *Case Report*. A 42-year-old female patient presented to our department nine weeks after a displaced distal humerus fracture, which was treated conservatively in another institution. Ulnar nerve neuropathy, pain, and severe stiffness of the elbow were her main symptoms. Open correction of the deformity, anatomical reduction of the articular surface, and realignment of the metaphyseal level of the fracture were performed. Six months postoperation, a painless flexion-extension arc of 110° and a normal rotation of the forearm was achieved.

**Conclusion:**

Distal humerus fracture malunion is a challenge. The operation needed for this purpose is much more demanding, and postsurgical complications are more likely to occur as opposed to the treatment of acute fractures. If a proper surgery is performed though, a good clinical outcome can be expected.

## 1. Introduction

Distal humerus fractures represent approximately the 2% of all fractures and the 30% of the fractures around the elbow [1]. They result either from a low-energy injury in the elderly or from a high energy injury in the young population. The aim of the management is to fix the distal humerus anatomically; otherwise, disability is inevitable [1]. The treatment is surgical, except for low-demand patients with comorbidities, which preclude general anesthesia. In this case, “a bag of bones” conservative treatment is recommended [2].

Open reduction and internal fixation of these fractures is always a challenge for the orthopaedic surgeon. Familiarity with the surgical approach and the various fixation methods and implants is required [2, 3]. Despite the evolution achieved regarding elbow surgery, the postoperative complication rates are reported as high as 35%, including mechanical failure, ulnar nerve palsy, stiffness, heterotopic ossification, nonunion, malunion, and infection [4].

Surgical management of malunited or nonunited distal humerus fractures is more challenging than acute fractures, but if it is performed properly, it results to a satisfactory functional result [5, 6]. Very few relative cases of neglected distal humerus fractures are reported in the literature, especially in young active patients, due to the fact that the majority of these injuries are now operated early. We present a case of a young female patient who has been treated in our institution for a malunited distal humerus fracture nine weeks post injury. A narrative review of the literature is also reported.

## 2. Case Presentation

**Figure 1:**
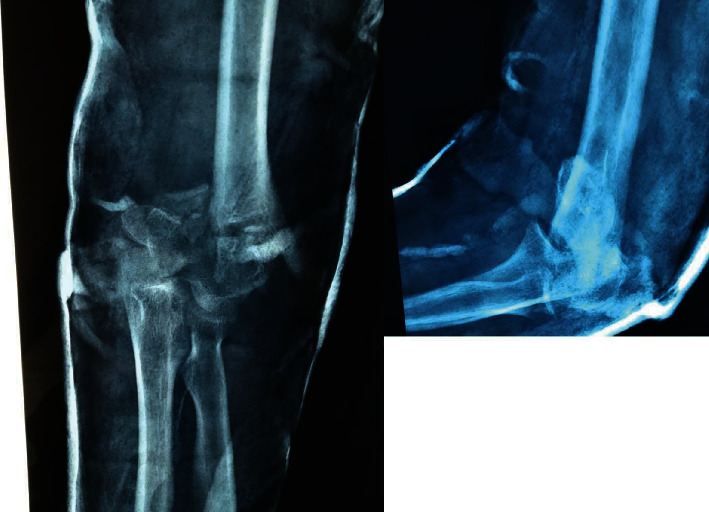
Distal humerus fracture malunion. Anteroposterior and lateral view.

A 42-year-old female patient presented to the clinics of our department with a malunited distal humerus fracture of the right dominant elbow, which took place approximately eight weeks before, after a fall from a height. The patient was treated conservatively in another hospital from abroad, where a splint was applied. She complained for a painful, stiff elbow, and ulnar nerve neuropathy symptoms. On examination, the ROM (range of motion) of the right elbow was 60° of extension to 90° of flexion (total sagittal motion arc of 30°), along with a very compromised rotation of the forearm. Paradoxical motion of the fracture area was identified as well. Reduced sensation of the ulnar two digits was also present. Plain X-rays were performed, revealing a type C2.1 distal humerus fracture, according to the AOOTA classification (Figure 1) [7]. Computed tomography (CT) scan demonstrated callus formation of the articular block and nonunion of the supracondylar part of the fracture.

**Figure 2:**
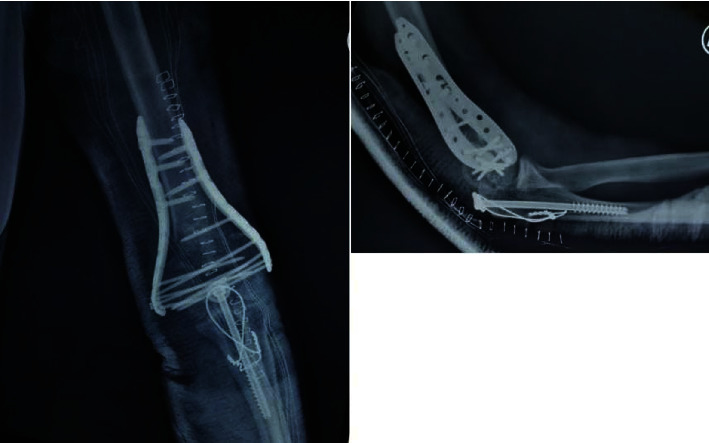
Postoperative radiographs of the elbow.

Taking into consideration the patient's age and the level of activity, surgical treatment was decided. The patient was applied into the lateral decubitus position and the elbow was held in a 90° of flexion. An extensile posterior longitudinal incision, curved on the olecranon tip, was performed. The ulnar nerve was identified, released, and transposed anterior to the medial epicondyle. A V type olecranon osteotomy was performed next, exposing the fracture. A malunited articular surface was noticed, along with a soft callus in the supracondylar part of the fracture. An oscillating saw and osteotome were used along the previous fracture lines, in order to mobilize the intraarticular fragments. Proper debridement of the soft callus was performed next, until healthy bleeding fragments were developed. After the fracture mobilization, K wires were used for temporary stabilization. Reduction was checked fluoroscopically. Definite osteosynthesis was performed using two parallel plates applied in a compression mode, with as many as possible distal screws engaging the opposite articular fragment, as per O' Driscoll's principles (Figure 2) [3]. A posterior elbow splint was applied for one week until the soft tissue edema and inflammation subsided. Physiotherapy was initiated afterwards.

**Figure 3:**
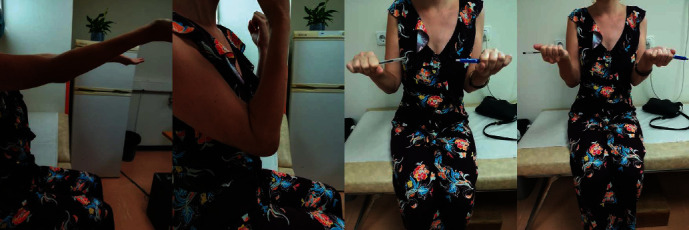
Right elbow ROM six months postoperation.

**Figure 4:**
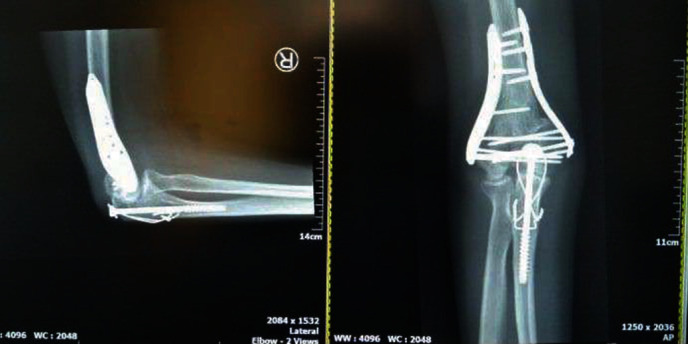
X-rays of the elbow six months postoperation.

On the final follow-up, 6 months postoperation, the patient scored 90 out of 100 points according to the MPI scoring system (Mayo Performance Index for the Elbow score) [8]. Only mild pain (visual analogue scale (VAS) for pain score: 1–2) was reported and the ROM of the elbow was 10°of extension to 120° of flexion, with a normal pronation and supination (Figure 3). The X-ray revealed complete fracture consolidation in a satisfactory position (Figure 4).

## 3. Discussion

Distal humerus fractures represent one of the most challenging injuries in orthopaedics, which they can result to a severe disability if not treated properly. Surgical fixation is almost always the treatment of choice. Conservative treatment can only be justified in cases of undisplaced extra-articular fractures or low-demand unhealthy patients, where general anesthesia cannot be given. The primary aim of fixation is to restore the distal humerus anatomy. Anatomical reduction and stable fixation of the fracture is required in order to achieve a painless functional elbow [9]. However, the complication rate is high [4]. Delayed operation for these injuries is much more demanding. Ulnar nerve release might be really difficult and copious due to soft callus or heterotopic bone. Fibrous tissue needs to be thoroughly removed and appropriate osteotomies along the previous fracture lines, if they exist, might be necessary, in order to recreate the fracture pattern and reduce it as anatomically as possible [5, 10, 11].

Thankfully, such injuries are not very common, because most of them are operated early. Very few cases of distal humerus fractures mal and nonunion are reported in the literature, and the majority of them have had an initial operation done. It is really odd that our case has been initially managed conservatively. Kinaci et al. [5] reported six cases of malunion which were treated with open reduction and internal fixation. Two of them developed neurologic impairment, one developed a deep infection and four of them needed hardware removal due to irritation. Overall, all patients were satisfied with the final result and the elbow ROM was significantly increased. Marti and Doornberg [11] reported on a case of a 48-year-old patient who was initially treated with a static external fixation, which led to unacceptable range of motion and pain. They performed an open reduction and internal fixation of the malunited fracture, 18 months post injury, achieving an excellent elbow ROM and functional score. McKee et al. [12] presented good functional results in 13 patients with malunited or nonunited distal humerus fractures, who were treated with open reconstruction of the elbow joint. Donders et al. [6] reported the largest series of 62 patients with distal elbow fracture malunion and/or nonunion, who were treated with operative joint reconstruction. 15 of them did not have any operation after the initial injury. New ulnar neuropathies were identified in five patients. Two patients developed superficial and two deep infection. One patient developed compartment syndrome and one median and radial nerve neuropathy. In most of the cases though, a painless useful elbow ROM was achieved. The fact that most of the distal humerus fractures are treated operatively and that the malunion high risk correction surgery is recommended in selected patients can explain why small relative case series are reported in the literature.

Distal humerus fracture mal or nonunion should be treated according to specific principles, as described by Jupiter and Vauclair et al. [10, 13]. An extensile longitudinal posterior incision is recommended. Olecranon osteotomy is the preferred method of approaching the fracture. If no signs of posttraumatic arthritis are evident and sufficient bone stock is available, then correction osteotomy and osteosynthesis, with or without bone grafts, is a viable option. Nonunited fragments should be debrided until well vascularized edges are produced. In case of a previous fixation, infection should be excluded. Stable fixation is of paramount importance for early mobilization [5, 10, 13]. The intraarticular level of the fracture should be fixed with as many screws as possible (at least three), which need to be inserted through the plate [3, 14]. Although parallel plate configuration seems to be more stable in biomechanical studies, than 90/90 fixation, this is not interpreted in vivo [9]. Total elbow arthroplasty is an option in low-demand elderly patients with poor bone stock and degenerative changes of the joint. Ilizarov type fixation is a useful tool for septic combined nonunions of the supracondylar and intracondylar part of the fracture [10, 13]. In our case, a good bone stock was preserved in a young healthy patient. Thus, open reduction of the intraarticular malunited level and debridement of the nonunited supracondylar level of the fracture, followed by a stable fixation, led to an excellent clinical outcome.

## 4. Conclusion

Open reduction and internal fixation is the gold standard of care for acute and chronic distal humerus fractures, provided that no excessive arthrosis is present. Mal and nonunions are a real challenge for the treating surgeon. Surgery is much more demanding and the complication rates are higher. Nevertheless, a painless and functional elbow can be expected if a proper surgical technique is performed.
